# Derivatives of Pyrimidine Nucleosides Affect Artificial Membranes Enriched with Mycobacterial Lipids

**DOI:** 10.3390/pharmaceutics16091110

**Published:** 2024-08-23

**Authors:** Olga S. Ostroumova, Svetlana S. Efimova, Polina D. Zlodeeva, Liudmila A. Alexandrova, Dmitry A. Makarov, Elena S. Matyugina, Vera A. Sokhraneva, Anastasia L. Khandazhinskaya, Sergey N. Kochetkov

**Affiliations:** 1 Institute of Cytology, RAS, Saint Petersburg 194064, Russia; 2 Engelhardt Institute of Molecular Biology, RAS, Moscow 119991, Russia

**Keywords:** antimycobacterial agents, pyrimidine nucleosides, lipid bilayers, mycolic acids, glycolipids, lipid melting, ion-permeable transmembrane pores

## Abstract

The mechanisms of action of pyrimidine nucleoside derivatives on model lipid membranes of various compositions were studied. A systematic analysis of the tested agents’ effects on the membrane physicochemical properties was performed. Differential scanning microcalorimetry data indicated that the ability of nucleoside derivatives to disorder membrane lipids depended on the types of nucleoside bases and membrane-forming lipids. The 5′-norcarbocyclic uracil derivatives were found to be ineffective, while N^4^-alkylcytidines demonstrated the most pronounced effects, significantly decreasing the dipalmitoylphosphocholine melting temperature and cooperativity of phase transition. The elongation of hydrophobic acyl radicals potentiated the disordering action of N^4^-alkylcytidines, while an increase in hydrophilicity due to replacing deoxyribose with ribose inhibited this effect. The ability of compounds to form transmembrane pores was also tested. It was found that 5-alkyluridines produced single, ion-permeable pores in phosphatidylglycerol membranes, and that methoxy-mycolic acid and trehalose monooleate potentiated the pore-forming activity of alkyloxymethyldeoxyuridines. The results obtained open up perspectives for the development of innovative highly selective anti-tuberculosis agents, which may be characterized by a low risk of developing drug resistance due to the direct action on the membranes of the pathogen.

## 1. Introduction

According to the World Health Organization (WHO), tuberculosis causes 1.5 million deaths every year. It also ranks among the top causes of death for HIV-infected people, and drug-resistant *Mycobacterium tuberculosis* heads the 2024 WHO Bacterial Priority Pathogens List. So, one of the main goals consists of a continuous, intensive search for new anti-tuberculosis agents, compounds that combine high antibacterial activity with decreased toxicity. Simultaneously with the clinical use of traditional anti-tuberculosis drugs such as bedaquiline, delamanid, linezolid, isoniazid, rifampicin, pyrazinamide, and ethambutol [1,2,3,4,5], searches for potential new anti-tuberculosis drug candidates, including SQ109 analogues and telacebec (Q203), have been performed [6,7]. Although dramatic progress has been achieved, an urgent need remains for new anti-tuberculosis drugs due to increasing pathogen resistance to available medications [1,8].

The unique structure and lipid composition of the cell envelope of mycobacteria, including an outer lipid layer containing long-chain mycolic acids and an inner plasma membrane enriched with anionic phospholipids, make it a major barrier for the penetration of drugs and largely contribute to pathogen survival. Successful therapeutic strategies have targeted the biosynthesis and transport of mycobacterial cell wall components, including the InhA and MmpL3 proteins, which participate in the biosynthesis and transport of mycolic acids [9,10]. This approach has several advantages. The unique structure of the mycobacterial cell wall and the enzymes involved in the biosynthesis of its components ensure the selectivity of the action of anti-tuberculosis drugs. However, serious disadvantages include the risk of developing resistance to the drugs targeting bacterial proteins. The most promising and valuable avenue to overcome multidrug resistance and shorten the duration of anti-tuberculosis treatment is the direct targeting of mycobacterial membrane structures and functions. Targeting mycolic acids is of particular interest because their levels are increased in resistant strains of mycobacteria compared to sensitive ones [11]. Several compounds with direct inhibitory effects on the function of mycobacterial membranes have been described in the literature. For instance, cationic amphiphilic indolyl Mannich bases have demonstrated activity against *M. bovis* BCG and *M. tuberculosis* H37Rv via membrane permeabilization and depolarization. Moreover, these agents have shown promising selectivity of action due to preferential disruption of bacterial membranes over mammalian ones. A role for compound lipophilicity in the antimycobacterial effect has been established [12]. Nitazoxanide and its active metabolite, tizoxanide, are membrane-active compounds that kill *M. tuberculosis* via multiple mechanisms, including the disruption of the bacterial membrane potential and alterations in the pH homeostasis [13,14]. The heterotricyclic xanthone α-mangostin restricts *M. tuberculosis* growth but has also demonstrated toxicity to mammalian cells. The antimycobacterial activity of α-mangostin derivatives involves a loss of membrane integrity in *M. bovis* [15]. Moreover, increasing xanthone lipophilicity improves bactericidal activity [16]. Boromycin is a potent inhibitor of *M. tuberculosis* growth due to its action as a potassium ionophore [17].

Despite the above data, it should be noted that the concept of directly damaging the mycobacterial membrane as a therapeutic strategy has not yet received enough attention. One of the reasons for the poor development of this area is the lack of realistic physical models of mycobacterial membranes. In the context of drug discovery (and the search for compounds capable of increasing the permeability of mycobacterial membranes in particular), understanding the biophysical properties of mycobacterial membranes is of crucial interest. Model lipid membranes can provide a unique toolkit for such studies, although very few reports in the literature have described the use of realistic physical models of mycobacterial membranes. Trehalose lipids can form hydrogen bonds with surrounding lipids, replacing water molecules on the surface of monolayers [18]. Adhyapak et al. have revealed the more dynamic and fluid behavior of outer membrane lipids compared to lipids from the inner membrane of *M. smegmatis* [19]. Langford et al. [20] have also described the pore-forming activity of MspA reconstituted into artificial, planar lipid membranes composed of mycolic acids extracted from *M. tuberculosis*. Using in silico modeling, Vasyankin et al. have demonstrated temperature-dependent phase transitions in mycolate membranes [21].

It has been shown recently that modified nucleosides are active against *M. tuberculosis* and *M. bovis* [22,23,24,25,26,27], and that the effects of some representatives of this class of compounds are related to damage to the mycobacterial cell wall [28]. However, the molecular mechanisms of the membrane-related action of these agents underlying their promising antimycobacterial activity should be further addressed.

The aim of this work was to elucidate the effects of pyrimidine nucleoside derivatives on model lipid membranes mimicking the outer and inner lipid membranes of mycobacteria. N^4^-alkylcytidines demonstrated pronounced effects on the thermotropic phase behavior of membrane lipids, and 5-alkyluridines were able to form ion-permeable pores in membranes enriched with phosphatidylglycerol, methoxy-mycolic acid, or trehalose monooleate.

## 2. Materials and Methods

**Figure 1:**
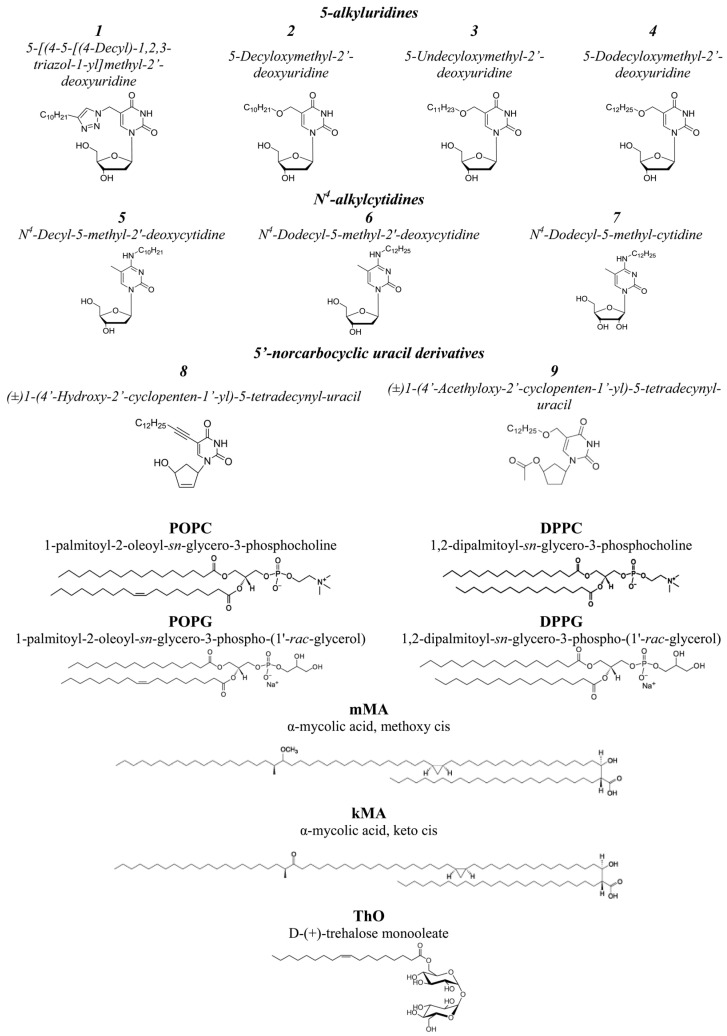
The chemical structures of tested nucleoside derivatives (***1***–***9***) and lipids used to construct membranes (phospholipids, mycolates and glycolipid).

All chemicals used in this study were of reagent grade. Synthetic 1-palmitoyl-2-oleoyl-*sn*-glycero-3-phosphocholine (POPC); 1-palmitoyl-2-oleoyl-*sn*-glycero-3-phospho-(1′-*rac*-glycerol) (POPG); 1,2-dipalmitoyl-*sn*-glycero-3-phosphocholine (DPPC); 1,2-dipalmitoyl-*sn*-glycero-3-phospho-(1′-*rac*-glycerol) (DPPG); α-mycolic acid, methoxy cis (mMA); α-mycolic acid, keto cis (kMA); and D-(+)-trehalose monooleate (ThO) were purchased from Avanti Polar Lipids (Avanti Polar Lipids, Inc., Alabaster, AL, USA). Calcein, Sephadex G-50, Triton X-100, KCl, EDTA, HEPES, dimethylsulfoxide (DMSO), and KOH were purchased from Sigma-Aldrich Company Ltd. (Merck KGaA, Darmstadt, Germany). Pyrimidine nucleoside derivatives ***1***–***9*** were synthesized according to previously published procedures [24,25,27]. The chemical structures of the antimycobacterial agents tested and the lipids employed are shown in Figure 1.

### 2.1. Differential Scanning Microcalorimetry of Lipid Phase Transitions

Differential scanning microcalorimetry experiments were performed using a μDSC 7EVO microcalorimeter (KEP Technologies, Setaram, Caluire-et-Cuire, France). Giant unilamellar vesicles composed of DPPC and DPPG were prepared by the electroformation method using Vesicle Pre Pro^@^ (Nanion Technologies GmbH, Munich, Germany) (standard protocol, 3 V, 10 Hz, 1 h, 55 °C). The resulting liposome suspension contained 3 mM lipid and was buffered by 5 mM HEPES at pH 7.4. Pyrimidine nucleoside derivatives ***1***–***9*** from stock solutions (50 mM in DMSO) were added to the liposome suspensions at lipid/agent molar ratios of 100:1, 50:1, 25:1, 10:1, and 5:1. The content of DMSO in the cuvette did not exceed 2.5 vol% and did not produce any changes in the lipid thermotropic behavior. The rates of heating and cooling of the liposomal suspensions were equal to 0.2 °C and 0.3 °C min^–1^, respectively. The reversibility of the thermal transitions was evaluated by reheating the sample immediately after the cooling step from the previous scan. The temperature dependence of the excess heat capacity was analyzed using Calisto Processing (KEP Technologies, Setaram, Caluire-et-Cuire, France).

DSC profiles were characterized by the presence of a pre-transition from the gel to ripple phase, the melting temperature related to the gel–fluid (main) transition (*T_m_*), and the enthalpy of the main phase transition (∆*H_cal_*). The temperature difference between the upper (onset) and lower (completion) boundary of the main phase transition (∆*T_b_*) was used to characterize the sharpness of the gel-to-liquid-crystalline phase transition. Calisto software 10.3 (Setaram, Caluire-et-Cuire, France) was applied to perform a decomposition analysis of the DPPC or DPPG main peaks in the presence of nucleoside derivatives. The separation of overlapping peaks was based on the application of Gaussian- and/or Fraser–Suzuki-type signals. The optimal parameters of each peak component, including the maximum temperature (*T_m___i_*, where *i* is the number of the component) and the percentage contribution of the distinct component to the total area/enthalpy of the main peak (Δ*H_i_*/Δ*H_cal_*), were estimated. Signal fitting was performed using a non-linear optimization (Marquardt, East Cazenovia, NY, USA) based on the deconvolution analysis of the main peak *T_m_* in the presence of nucleoside derivatives and was calculated as $T_{m}=\sum_{i}T_{m\_i}\frac{\Delta H_{i}}{\Delta H_{cal}}$. Two to four independent experiments were performed with each experimental system to obtain the *T_m_*, Δ*H_cal_*, and Δ*T_b_* values.

### 2.2. Preparation of Planar Lipid Bilayers and Recording of Current

Virtually solvent-free planar lipid bilayers were prepared according to a monolayer-opposition technique [29] on a 50 µm diameter aperture in a 10 µm thick Teflon film separating two (*cis* and *trans*) compartments of a Teflon chamber. The aperture was pretreated with hexadecane. Lipid bilayers were created from pure POPC or POPG and mixtures of POPG/mMA, POPG/kMA, and POPG/ThO (80/20 mol%). The membrane-bathing solution was 0.1 M KCl buffered by 10 mM HEPES at pH 7.4. After the planar lipid bilayer had been formed and stabilized, compounds ***1***–***9*** from the stock solutions (25 mM in DMSO) were added to the aqueous solution at the *cis* side of the chamber to obtain a concentration of less than 450 μM. DMSO at used concentrations (corresponding to the minimum and maximum solvent volumes that were used when adding various agents to the membrane bathing solution to reach their threshold pore-forming and detergent concentrations) did not alter the stability and ionic permeability of the lipid bilayers of different compositions in the absence of the tested compounds ***1***–***9*** (Supplementary Figure S1).

Ag/AgCl electrodes with agarose/2 M KCl bridges were used to apply the transmembrane voltage (*V*) and to measure the transmembrane current. “Positive voltage” refers to a case in which the *cis*-side compartment was positive with respect to the *trans* side. All experiments were performed at room temperature. Current measurements were performed using an Axopatch 200B amplifier (Molecular Devices, LLC, Orleans Drive, Sunnyvale, CA, USA) in voltage-clamp mode. The data were digitized by Digidata 1440A and analyzed using pClamp 10 (Molecular Devices, LLC, Orleans Drive, Sunnyvale, CA, USA) and Origin 7.0 (OriginLab Corporation, Northampton, MA, USA). Current tracks were filtered by an eight-pole Bessel at 100 kHz.

The effect of compounds ***1***–***9*** on the ionic permeability of model lipid membranes of different compositions was characterized by the threshold concentration at which step-like transmembrane current fluctuations related to the opening and closing of single ion-permeable pores were observed (*C_sc_*), the threshold concentration disrupting the lipid bilayer at the transmembrane voltage of 100 mV (*C_tr_*), and the range of conductance of single ion-permeable pores (*g_sc_*).

The values of *C_sc_*, *C_tr_*, and *g_sc_* were averaged from three to four independent experiments and presented as mean ± standard error (*p* ≤ 0.05).

### 2.3. Calcein Release from Unilamellar Lipid Vesicles

The fluorescence of calcein leaked from unilamellar lipid vesicles was used to monitor the membrane permeabilization induced by compounds ***1***–***9***. Liposomes loaded with calcein buffer (35 mM calcein, 10 mM HEPES–KOH, pH 7.4) were made from pure POPC or POPG by extrusion using an Avanti Polar Lipids mini-extruder with a 100 nm membrane (Avanti Polar Lipids, Inc., Alabaster, AL, USA). After gel-filtration, calcein-loaded liposomes were resuspended in 0.15 M KCl with 1 mM EDTA and 10 mM HEPES at pH 7.4. Markers inside the vesicles fluoresced very poorly due to strong self-quenching at mM concentrations, while the fluorescence of disengaged calcein in the surrounding medium correlated to membrane permeabilization induced by the tested pyrimidine nucleoside derivative.

The degree of calcein release was determined at 25 °C using a Fluorat-02-Panorama spectrofluorimeter (Lumex, Saint-Petersburg, Russia). The excitation wavelength was 490 nm, and the emission wavelength was 520 nm. The detergent Triton X-100 (at a final concentration of 1%) was added at the end of the experiments to produce complete disruption of liposomes (referring to full disengagement of the marker from vesicles).

The relative intensity of calcein fluorescence (*IF*, %) was used to describe the dependence of the permeabilization of the liposomes on the type of compound. *IF* was calculated using the following formula:

(1) $$IF=\frac{I-I_{0}}{I_{\max}/0.9-I_{0}}\cdot 100\%$$

where *I* and *I_0_* are the calcein fluorescence intensities in the sample in the presence and absence of nucleoside derivatives ***1***–***9***, respectively, and *I_max_* is the maximal fluorescence of the sample after lysis of the liposomes by Triton X-100. A factor of 0.9 was introduced to account for the dilution of the sample by detergent.

The values of *IF* were averaged from two to three independent experiments and have been presented as mean ± standard error (*p* ≤ 0.05).

## 3. Results and Discussion

### 3.1. Effects of Pyrimidine Nucleoside Derivatives on Lipid Packing

Differential scanning microcalorimetry was used to assess the effects of the tested derivatives on membrane lipid packing. Two model systems were used to mimic the membranes of mammalian cells and the inner membrane of mycobacteria. The first was composed of DPPC because phosphatidylcholines are most abundant in mammalian membranes [30,31]. The second was composed of DPPG because the membranes of all Gram-positive bacteria and the inner membranes of Gram-negative bacteria are enriched with phosphatidylglycerols [32,33].

**Figure 2:**
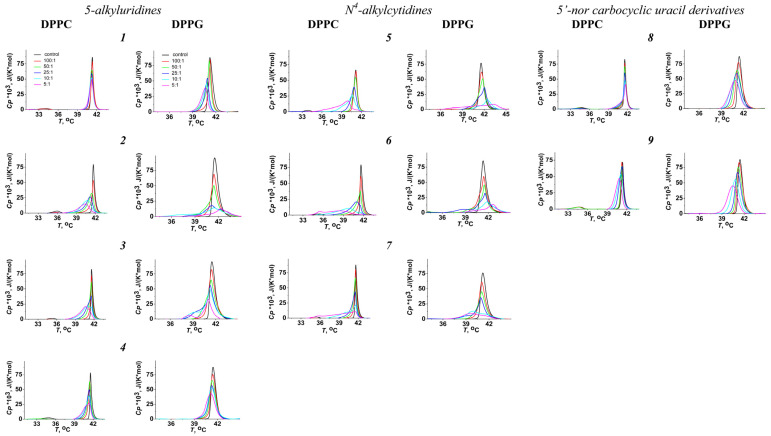
Heating thermograms of DPPC and DPPG liposomes in the absence (*black lines*) and presence of various nucleoside derivatives at the lipid-to-agent ratio of 100:1 (*red curves*), 50:1 (*blue curves*), 25:1 (*green curves*), 10:1 (*cyan curves*), and 5:1 (*purple curves*).

Figure 2 presents the DPPC and DPPG heating thermograms in the absence (*control, black curves*) and presence of compounds ***1***–***9*** in the liposome suspensions at lipid-to-agent ratios of 100:1 (*red curves*), 50:1 (*blue curves*), 25:1 (*green curves*), 10:1 (*cyan curves*), and 5:1 (*purple curves*). In the absence of any agents, the pre-transition temperature of DPPC, *T_p_*, was 32.6 ± 0.2 °C, and the main phase transition temperatures, *T_m_*, were 41.5 ± 0.1 and 41.3 ± 0.2 °C for DPPC and DPPG, respectively. The widths of the main peaks, characterizing the sharpness of the melting, ∆*T_b_*, were 2.0 ± 0.1 °C and 2.7 ± 0.4 °C for DPPC and DPPG, respectively. The results obtained with pure lipids (concerning the transition temperatures and enthalpy) were in a good agreement with the literature data [34,35]. The pre-transition of DPPC, which was related to the transition from the gel to the intermediate-ripple phase, was suppressed by the nucleoside derivatives (at least at low lipid/agent ratios), with the exception of compound ***8*** (Figure 2).

In the presence of the tested agents, the main peaks on the thermograms of DPPC and DPPG became asymmetric (Figure 2). Supplementary Figure S2 clearly demonstrates that the main peaks on the DPPC thermograms in the presence of the N^4^-alkylcytidine compounds ***5***, ***6***, and ***7*** (at least at the lipid/agent ratios of 10:1 and 5:1) have a complex nature and may have been deconvoluted for two or three components with various melting temperatures (Supplementary Table S1). Less-pronounced deconvolution of the DPPC main peak was observed after the addition of compounds ***4*** and ***8***. The main peak of DPPG could be decomposed on two or three overlapping transitions in the presence of not only the N^4^-alkylcytidine compounds ***5***, ***6***, and ***7*** but also the 5-alkyluridine compounds ***2*** and ***3*** at lipid/agent ratios of 25:1, 10:1, and 5:1 (Supplementary Figure S2, Supplementary Table S1). Strong profile consistency from repeated heating steps demonstrated that the complex nature of the main peak was not due to a non-equilibrium distribution of derivatives between lipid monolayers. Therefore, this observation more probably indicated the existence of several lipid phases enriched with different concentrations of the nucleoside derivative. Moreover, the contribution Δ*H_i_*/Δ*H_cal_* of the low-melting components increased with increasing concentrations of N^4^-alkylcytidines, while the contribution of the high-melting components decreased (Supplementary Table S1). Phase separation within the 1,2-dimyristoyl-*sn*-glycero-3-phosphocholine bilayers was also proposed by Escobar et al. [36,37] in the presence of a sterol–uridine conjugate and tri-acyl ester derivatives of uridine nucleoside, based on the analysis of the DSC thermograms (the appearance of shoulders on the main peak as a consequence of a non-ideal mixing behavior and nonhomogeneous distribution of the compound within the membranes indicating the existence of several lipid phases, enriched and poor, in the compound). Compound ***1***, with a triazol linker between a decyl radical and methyldeoxyuridine, and the acethylated uracil derivative **9** did not convert the main peaks of DPPC and DPPG into a multicomponent profile. To account for the existence of several lipid phases with different melting points in the presence of some derivatives, we calculated the mean normalized melting temperature as $T_{m}=\sum_{i}T_{m\_i}\frac{\Delta H_{i}}{\Delta H_{cal}}$. Using the described approach allowed us to compare the effects of different pyrimidine nucleoside derivatives.

**Figure 3:**
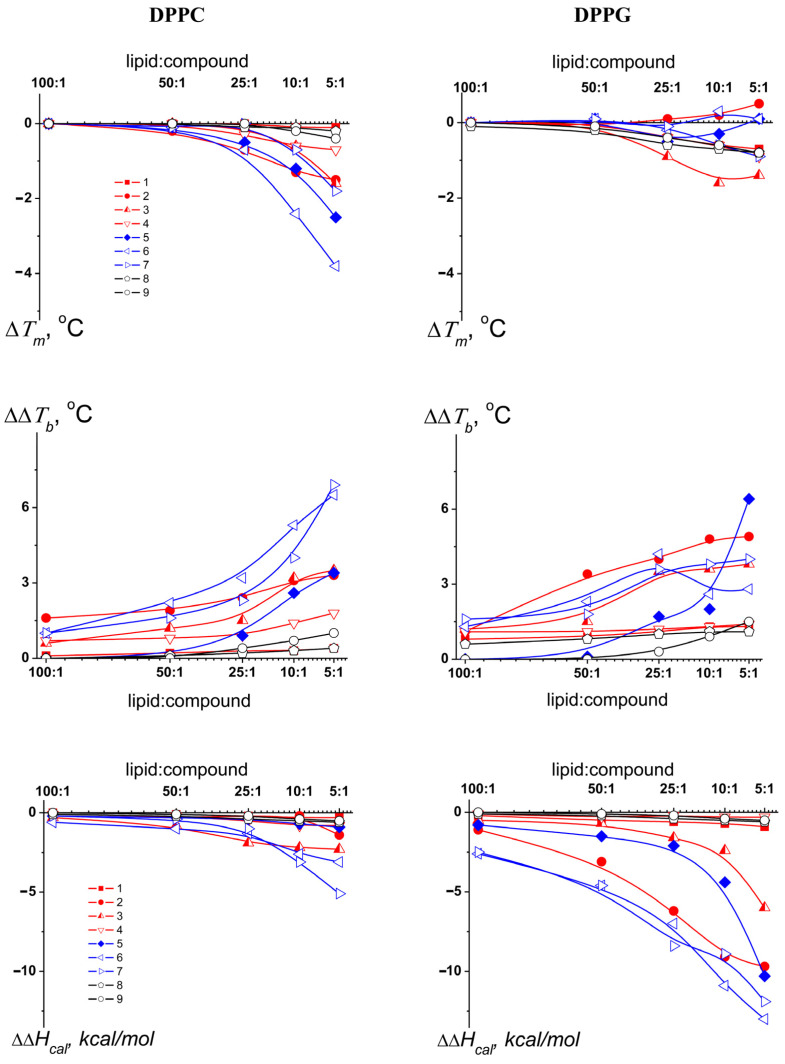
The dependence of the changes in the melting temperature (Δ*T_m_* *, upper panel), in the width of main peak on the thermogram (ΔΔ*T_b_*, middle panel), and in the enthalpy of the main transition (ΔΔ*H_cal_*, lower panel) of DPPC (left column) and DPPG (right column) on the lipid/compound molar ratio. * In the presence of nucleoside derivatives *T_m_* was calculated based on the deconvolution analysis of the main peak as $T_{m}=\sum_{i}T_{m\_i}\frac{\Delta H_{i}}{\Delta H_{cal}}$ (Supplementary information).

Figure 3 presents the dependence of the difference between the mean normalized melting temperature in the presence of the nucleoside derivative and the control value of *T_m_* for pure lipid on the lipid/compound ratio. It is noteworthy that the derivative effect on DPPC melting temperature strictly depended on the nucleoside base; the 5′-norcarbocyclic uracil derivatives were ineffective, and the 5-alkyluridines had a weak effect (with the exception of the compound ***1***, which had no effect) on the thermotropic characteristics of DPPC, while the N^4^-alkylcytidines demonstrated the most pronounced effects, significantly decreasing the melting temperature of DPPC. As a rule, a decrease in the melting point occurs as a result of the incorporation of membrane-active compounds between lipid heads and a subsequent increase in the area per lipid molecule and the mobility of lipid acyl chains. The integration of agents between lipids is usually accompanied by a decrease in the cooperativity of the phase transition, which results in an increase in the width of the peak corresponding to melting. Accordingly, a similar dependence on the type of nucleoside base was also observed in relation to ΔΔ*T_b_* values (Figure 3). All these effects are usually described in terms of a disordering or uncoupling action of the agents on the lipid bilayer. An increase in the content of 5-alkyluridines ***2***–***4*** and N^4^-alkylcytidines ***5***–***7*** (up to a molar ratio of 5:1) caused a significant increase in *T_m_*-hysteresis (the difference in the transition temperature between heating and cooling scans) (by 0.5–1.8 °C) (Supplementary Figure S3). The phenomenon of hysteresis is associated with the reversibility and kinetics of the formation, growth, and optimization of liquid-crystalline or gel domains in gel or fluid phase, respectively [38]. Derivatives ***2*** and ***6*** were characterized by the greatest ability to increase *T_m_*-hysteresis, and the effect of these compounds on the freezing temperature was significantly greater than on the melting temperature (by 2 and 1 °C, respectively). This might indicate a stronger interaction of 5-alkyluridine ***2*** and N^4^-alkylcytidine ***6*** with the liquid-crystalline lipid phase than with the gel state. Moreover, the inhibition of the formation of ordered lipid clusters during the liquid to gel transformation upon cooling by the compounds was consistent with the assumption of their marked disordering action. From this consideration, it becomes clear why the elongation of the alkyl radical, linked to methyldeoxycytidine, from decyl (in compound ***5***) to dodecyl (in compound ***6***) (which led to increase in logarithm of octanol–water partition coefficient, *LogP* (Table 1)) enhanced the effects on *T_m_* and Δ*T_b_* (Figure 3). An increase in the length of the hydrophobic chain led to a deeper immersion of the derivative into the lipid bilayer, and the disordering effect therefore became more pronounced. Notably, this pattern did not hold for 5-alkyluridines. The acyltriazol derivative of methyldeoxyuridine (compound ***1***) did not appreciably affect DPPC melting (Figure 3). Replacement of the triazole motif (in compound ***1***) with an ether linkage (in compound ***2***) enhanced the effects of the derivative on DPPC thermotropic behavior, as a dose-dependent decrease in *T_m_* and an increase in Δ*T_b_* were observed (Figure 3). Elongation of the alkyl radical from decyl (in compound ***2***) to undecyl (in compound ***3***) had no noticeable effect on the ability of the alkyl derivative of oxymethyldeoxyuridine to modify the phase transition of DPPC (Figure 3). Surprisingly, a further increase in the length of the side radical from undecyl (in compound ***3***) to dodecyl (in compound ***4***) resulted in a decrease in the effects on *T_m_* and Δ*T_b_* (Figure 3). This effect may indicate that the derivative with the longest “tail” acquired a different orientation of its nucleoside “head” in the lipid bilayer compared to compounds with shorter hydrocarbon chains.

It is clear that a decrease in the hydrophobicity of a compound (Table 1) should reduce its immersion in the membrane and its effects on the mobility of the lipid acyl chains, that is, on the melting temperature. At a constant length of the hydrophobic radical, the addition of an extra hydroxyl group to a sugar residue of N^4^-alkylcytidine (replacement of deoxyribose [in compound ***6***] for ribose [in compound ***7***]), as expected, reduced the disordering effect of the derivative (Figure 3).

The effects of the tested compounds on the melting temperature of the negatively charged DPPG were weaker than those of the neutral DPPC (Figure 3). Only the undecyl derivative of methyldeoxyuridine (compound ***3***) demonstrated the ability to decrease *T_m_* independently of the charge of the membrane-forming lipids (Figure 3). Notably, 5-alkyluridines ***2*** and ***3*** and all the tested N^4^-alkylcytidines significantly decreased the sharpness and enthalpy of the DPPG transition (Figure 3). In this regard, the observed increase in the width of the peak corresponding to the melting of DPPG (Δ*T_b_*) may indicate their sorption on the surface of negatively charged membranes, while a decrease in Δ*H_cal_* may be related to the formation of nonlamellar DPPG phase in the presence of the compounds. Taking into account the significantly smaller changes in the transition enthalpy of DPPC in the presence of pyrimidine nucleoside derivatives (Figure 3), it was unlikely that they induced lamellar–non-lamellar transformations of neutral lipids.

### 3.2. Effects of Nucleoside Derivatives on Membrane Permeability

#### 3.2.1. Phospholipid Bilayers

The ability of nucleoside derivatives to form ion-permeable transmembrane pores in planar lipid bilayers composed of POPC or POPG was also studied. Table 1 presents the threshold concentrations that caused the appearance of single-step-like fluctuations in the transmembrane current (*C_sc_*) and destruction of the lipid bilayers (*C_tr_*).

**Figure 4:**
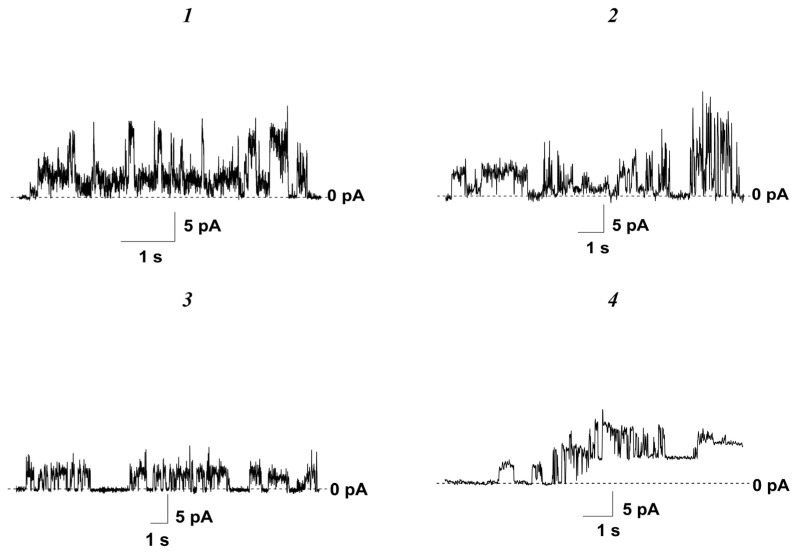
Typical records of step-like fluctuations in the transmembrane current induced by *cis*-side addition of compounds ***1***, ***2***, ***3***, and ***4*** at the concentration of 180, 150, 140, and 160 μM, respectively. Membranes were composed of POPG and bathed in 0.1 M KCl (pH 7.4). The transmembrane voltage was equal to 100 mV.

The introduction of all tested nucleosides up to concentrations of less than 370 ± 35 μM in the solution bathing the POPC membranes did not cause an increase in the bilayer ion permeability at a transmembrane voltage of 100 mV (Table 1). A further increase in the compound concentration led to the disruption of the electrical stability of membranes and their disintegration. Replacement of the neutral membrane-forming lipid POPC for the negatively charged lipid POPG resulted in a 1.2–1.7-fold decrease in the *C_tr_* of nucleoside derivatives ***1***–***7*** (Table 1). The threshold concentrations of the 5′-norcarbocyclic uracil derivatives ***8*** and ***9*** for disrupting the POPG bilayers were close to the *C_tr_* values characterizing the effects on the POPC membranes (Table 1). Surprisingly, the 5-alkyluridine compounds ***1****–**4*** were capable of inducing step-like fluctuations in the current through the POPG membranes at subthreshold concentrations (Table 1). Figure 4 presents the typical traces of transmembrane current fluctuations related to the openings and closures of transient single ion-permeable pores induced by derivatives ***1****–**4*** in the POPG bilayers bathed in 0.1 M KCl at a transmembrane voltage of 100 mV. The amplitude of the observed step-like current fluctuations induced by the 5-alkyluridine compounds ***1****–**3*** in POPG membranes varied in the range of several picoamperes, while compound ***4*** produced current fluctuations in the range of tens of picoamperes at 100 mV (Table 1). The number of open pores did not increase with an increase in the concentration of 5-alkyluridines. Other tested compounds related to N^4^-alkylcytidines and 5′-norcarbocyclic uracils did not exhibit pore-forming activity.

At 10 µM, N^4^-alkylcytidines and 5′-norcarbocyclic uracil derivatives did not induce a significant calcein release from the liposomes composed of POPC and POPG (the maximum value of relative fluorescence [*IF_max_*] characterizing the compound-produced increase in membrane permeability for calcein did not exceed 14%) (Table 1). The ability of 5-alkyluridines to disengage calcein from the lipid vesicles depended on their composition; the 5-alkyluridines that were tested induced significant leakage of the fluorescent marker from POPG vesicles, and this ability was weakened in the order of compound ***1*** ≥ compound ***2*** ≈ compound ***4*** > compound ***3*** (Table 1). The more pronounced ability of 10-µM 5-alkyluridines to disengage calcein from the vesicles containing POPG (*IF_max_* = 30–50%) (Table 1) is consistent with their unique ability to form ion-permeable pores in POPG membranes (Figure 4, Table 1). This effect may indicate that the pores formed by compounds ***1***–***4*** in POPG membranes were characterized by a relatively large size suitable for the transport of not only ions but also the fluorescent marker. A two-fold increase in the concentration of agents resulted in full disengagement of entrapped calcein, independently of membrane lipid composition and nucleoside type (*IF_max_* was about 100%). At this concentration, the derivatives were probably already characterized by a significant detergent effect, leading to the full destruction of the membranes.

#### 3.2.2. Lipid Bilayers Enriched with Specific Mycobacterial Lipids

Notably, 5-Alkyl-2′-deoxyuridines (compounds ***1***, ***3***, and ***4***) and 5′-norcarbocyclic uracils (compounds ***8*** and ***9***) demonstrated promising activity against *M. tuberculosis* (H37Rv), with the concentrations exhibiting 99% inhibition of growth (MIC_99_) of 10–20 µg/mL [24,25,26]. The N^4^-alkyl-2′-deoxycytidine compounds ***5*** and ***6*** were shown to inhibit the growth of *M. smegmatis* (mc^2^155 and VKPM Ac-1339), with the respective MIC_99_ values of 50 and 24 µg/mL, and the N^4^-alkylcytidine compound ***7*** inhibited the growth of *M. smegmatis* (mc^2^155 and VKPM Ac-1339), with an MIC_99_ of 32 µg/mL [27,39]. The independence of the MIC_99_ values from the type of *M. tuberculosis* strain (sensitive laboratory strain H37Rv versus resistant clinical strain MS-115) for compounds ***1*** and ***8***, in contrast to well-known anti-tuberculosis drugs such as rifampicin, isoniazid, ethambutol, and pyrazinamide (which are ineffective against MS-115) [24,25], confirms an alternative mode of action of alkyl derivatives of pyrimidine nucleosides related to the direct disturbance of mycobacterial membranes. Considering this, specific mycobacterial lipids, in particular methoxy- and keto-mycolic acids (Figure 1), were introduced into the bilayer composition.

The addition of 20 mol% mMA into the POPG bilayer led to a dramatic decrease in the *C_tr_* of the tested nucleoside derivatives ***1***, ***2***, ***4***, ***6***, ***8***, and ***9*** (by 1.5–17 times) (Table 2). The disordering activity of the 5-alkyluridine compound ***1*** (with a triazole motif) was not affected by the mycolic acid composition of the bilayer, whereas the effects of the 5-alkyloxymethyl-2′-deoxyuridine compounds ***2*** and ***4*** were strictly dependent on the type of mycolic acid in the membrane. The concentrations of compounds ***2*** and ***4*** causing the disintegration of POPG/kMA (80/20 mol%) bilayers were similar to or even higher than those for membranes composed of pure POPG and were significantly higher than those for the POPG/mMA bilayers. The ability of the N^4^-alkylcytidine compound ***6*** to cause membrane collapse was not significantly influenced by the type of mycolic acid. Similar to mMA, the inclusion of kMA into the POPG bilayers produced a reduction in the *C_tr_* of the 5′-norcarbocyclic uracil derivatives ***8*** and ***9***. The *C_tr_* of derivative ***9*** did not significantly depend on the type of mycolic acid in the POPG membranes, whereas the introduction of kMA instead of mMA in the bilayer’s composition led to an alteration in the *C_tr_* of the 5′-norcarbocyclic uracil derivative ***8***.

**Figure 5:**
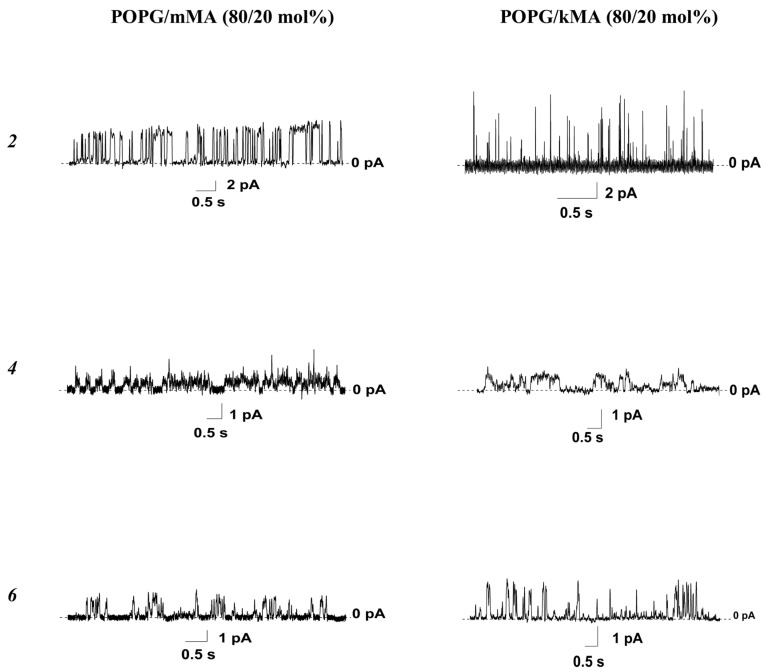
Typical records of step-like fluctuations in the transmembrane current induced by *cis*-side addition of compounds ***2***, ***4***, and ***6*** at the concentration of 25 μM (or 30 μM), 15 μM (or 20 μM), and 5 μM (or 7 μM), respectively. Membranes were composed of POPG/mMA (80/20 mol%) (*left panel*) or POPG/kMA (80/20 mol%) (*right panel*) and bathed in 0.1 M KCl (pH 7.4). The transmembrane voltage was equal to 100 mV.

Contrary to its effect on the membranes composed of pure POPG (Figure 4, Table 1), the 5-alkyluridine compound ***1***, with a triazole motif, was not able to produce step-like current fluctuations in the membranes containing mycolic acids (Table 2). The concentrations of the 5-alkyloxymethyl-2′-deoxyuridine compounds ***2*** and ***4*** at which single ion-permeable pores were observed (*C_sc_*) in the POPG/mMA membranes were lower than those for the bilayers composed of pure POPG. A type of mycolic acid did not affect the *C_sc_* of compounds ***2*** and ***4***. Surprisingly, the N^4^-alkylcytidine compound ***6*** was able to induce step-like fluctuations in the current through both the POPG/mMA and POPG/kMA bilayers at extremely low concentrations, whereas it did not produce such activity in membranes composed of pure POPG. Figure 5 presents the typical traces of transmembrane current fluctuations related to functioning transient single ion-permeable pores induced by compounds ***2***, ***4***, and ***6*** in the POPG bilayers containing various mycolic acids. The 5′-norcarbocyclic uracil derivatives ***8*** and ***9*** were unable to produce ion-permeable pores in the bilayers containing both mMA and kMA as well as in the membranes composed of pure POPG.

**Figure 6:**
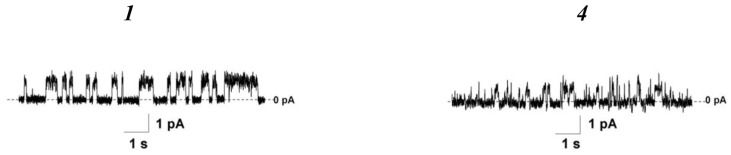
Typical records of step-like fluctuations in the transmembrane current induced by *cis*-side addition of compounds ***1***, and ***4*** at the concentration of 5 and 15 μM, respectively. Membranes were composed of POPG/ThO (80/20 mol%) and bathed in 0.1 M KCl (pH 7.4). The transmembrane voltage was equal to 100 mV.

Mycolic acids in the cell walls of mycobacteria are often associated with trehalose, forming trehalose monomycolates and dimycolates [40]. To estimate the possible impact of trehalose-containing glycolipids, we tested the effects of the 5-alkyluridine compounds ***1*** and ***4*** on lipid bilayers enriched with trehalose monooleate (ThO). The threshold concentrations of compounds ***1*** and ***4*** causing the destruction of the POPG/ThO (80/20 mol%) bilayers were 7–9-fold lower than those for the membranes composed of pure POPG and 4–5-fold lower than those for the POPG/mMA bilayers (Table 2). Moreover, both agents were able to produce ion-permeable pores in the POPG/ThO membranes (Figure 6, Table 2). Thus, we concluded that the introduction of mMA and ThO into the POPG bilayers had potentiated the membrane action of these 5-alkyluridines. The tested 5′-norcarbocyclic uracil derivatives did not exhibit pore-forming activity in bilayers containing ThO. In addition, the concentration of compound ***8*** causing the disintegration of POPG/ThO bilayers was similar to or even higher than that for the membranes made from pure POPG or the mixtures of POPG and mycolic acids (Table 2). The inclusion of ThO in the bilayer composition also facilitated the disintegrating activity of derivative ***9***.

Summarizing the data obtained with mycobacterial-like lipids, we postulate the following:

(1) Addition of mMA into POPG bilayers potentiated the disintegrating activity of all tested nucleoside derivatives and the pore-forming ability of compounds ***2***, ***4***, and ***6***; it also inhibited the induction of ion-permeable pores by the 5-alkyluridine compound ***1***;
(2) Introduction of kMA into POPG membranes facilitated the disordering activity of derivatives ***1***, ***6***, ***8***, and ***9*** and the pore-forming ability of compounds ***2***, ***4***, and ***6***;
(3) Inclusion of ThO into POPG bilayers led to an increase in the ability of 5-alkyluridines ***1*** and ***4*** to cause membrane collapse and form ion-permeable pores, as well as inhibit and potentiate the disintegrating action of compounds ***8*** and ***9***, respectively.

## 4. Conclusions

In this work, we have developed models of the inner and outer membranes of mycobacteria enriched with anionic phospholipids and mycolic acids, respectively. We have shown for the first time that the antimycobacterial activity of pyrimidine nucleosides may be associated with their disordering effect on the inner membranes of mycobacteria, as well as on pore formation in the inner and outer membranes. In particular, N^4^-alkylcytidines significantly affected membrane lipid packing, while 5-alkyluridines produced ion-permeation defects. We highlight that this is the first direct evidence of the formation of drug-induced pores in mycobacterial-like membranes. Our results have provided significant insights into the molecular details of the action of the tested compounds on target membranes, structure–activity relationships, and the influence of lipid composition on the agents’ activity. The action of these compounds has been shown to be strictly dependent on membrane lipid composition, the presence of anionic phospholipids, glycosylated lipids, and various mycolic acids, opening the way to developing innovative anti-tuberculosis formulations.

## Figures and Tables

**Table 1:** Parameters charactering the effects of nucleoside derivatives on the permeability of bilayers composed of POPC or POPG for ions and fluorescent marker.

*Compound*	*LogP*	POPC		POPG			
		*C_tr_*, μM	*IF_max_*, %	*C_tr_*, μM	*C_sc_*, μM	*g_sc_*, pS	*IF_max_*, %
***1***	1.92	355 ± 10	8 ± 4	220 ± 15	175 ± 10	5–40	47 ± 5
***2***	1.86	360 ± 15	5 ± 3	250 ± 10	140 ± 15	5–10	38 ± 7
***3***	2.26	375 ± 10	10 ± 3	240 ± 15	145 ± 10	5–50	26 ± 4
***4***	2.66	350 ± 25	18 ± 4	210 ± 10	150 ± 10	30–210	39 ± 3
***5***	3.14	375 ± 10	7 ± 3	275 ± 15	– ^#^	– ^#^	5 ± 3
***6***	3.93	405 ± 15	6 ± 2	280 ± 10	– ^#^	– ^#^	11 ± 4
***7***	3.02	340 ± 20	5 ± 3	295 ± 15	– ^#^	– ^#^	10 ± 5
***8***	4.58	355 ± 10	4 ± 2	350 ± 10	– ^#^	– ^#^	14 ± 3
***9***	4.00	380 ± 20	3 ± 1	340 ± 15	– ^#^	– ^#^	4 ± 2

*LogP* is the logarithm of the octanol–water partition coefficients of tested compounds estimated using the InstantJChem 18.8.0 program (ChemAxon, https://chemaxon.com); *C_tr_* is the threshold concentration of the agent at which destruction of the lipid bilayer was observed at the transmembrane voltage 100 mV; *C_sc_* is the threshold concentration of the agent at which step-like fluctuations in the transmembrane current, corresponding to the opening/closing of single transient ion-permeable transmembrane pores, were observed; *i_sc_* is the amplitude of fluctuations in the transmembrane current flowing through single ion-permeable pores produced by tested compounds; *IF_max_* is a maximum leakage of fluorescent marker from liposomes at compound concentration of 10 µM. #—this type of activity was not observed.

**Table 2:** Parameters characterizing the effects of nucleoside derivatives on the ion permeability of bilayers composed of POPG and various mycolic acids (mMA or kMA) or trehalose monooleate (ThO).

	POPG/mMA (80/20 mol%)			POPG/kMA (80/20 mol%)			POPG/ThO (80/20 mol%)		
*Compound*	*C_tr_*, μM	*C_sc_*, μM	*g_sc_*, pS	*C_tr_*, μM	*C_sc_*, μM	*g_sc_*, pS	*C_tr_*, μM	*C_sc_*, μM	*g_sc_*, pS
***1***	80 ± 30	– ^#^	– ^#^	83 ± 28	– ^#^	– ^#^	20 ± 10	8 ± 3	5–24
***2***	105 ± 55	58 ± 32	10–40	250 ± 50	65 ± 35	5–100	*nt* *	*nt* *	*nt* *
***4***	105 ± 73	22 ± 12	5–20	300 ± 50	20 ± 3	5–50	23 ± 3	18 ± 5	5–50
***6***	16 ± 7	3 ± 1	5–100	33 ± 18	9 ± 2	5–70	*nt* *	*nt* *	*nt* *
***8***	21 ± 4	– ^#^	– ^#^	90 ± 7	– ^#^	– ^#^	525 ± 140	– ^#^	– ^#^
***9***	62 ± 10	– ^#^	– ^#^	44 ± 12	– ^#^	– ^#^	80 ± 25	– ^#^	– ^#^

#—this type of activity was not observed; *—not tested.

## Data Availability

Data are contained within the article.

## Supplementary Materials

The following are available online at https://www.mdpi.com/article/10.3390/pharmaceutics16091110/s1, Supplementary Figure S1. The effects of different volumes of DMSO on the ionic permeability of POPC (a), POPG (b), POPG/kMA (80/20 mol%) (c) and POPG/ThO (80/20 mol%) (d) bilayers bathed in 0.1 M KCl pH 7.4. The transmembrane voltage was 100 mV. Arrows indicate the moments of addition of the DMSO into the membrane bathing solution. Supplementary Figure S2. Deconvolution analysis of the main peak on DPPC and DPPG thermograms in the presence various nucleoside derivatives at the lipid-to-agent ratio of 25:1, 10:1 and 5:1. The parameters characterizing distinct components are summarized in Supplementary Table S1. Supplementary Figure S3. The dependences of *T_m_*-hysteresis of DPPC on the lipid/compound molar ratio. The dotted line represents the control level of *T_m_*-hysteresis, which was observed in the absence of nucleoside derivatives. Supplementary Table S1. The main peak decomposition analysis performed in the presence of nucleoside derivatives.

## Abbreviation

POPC—1-palmitoyl-2-oleoyl-*sn*-glycero-3-phosphocholine, POPG—1-palmitoyl-2-oleoyl-*sn*-glycero-3-phospho-(1′-*rac*-glycerol), DPPC—1,2-dipalmitoyl-*sn*-glycero-3-phosphocholine, DPPG—1,2-dipalmitoyl-*sn*-glycero-3-phospho-(1′-*rac*-glycerol), mMA—α-mycolic acid, methoxy cis, kMA—α-mycolic acid, keto cis, ThO—D-(+)-trehalose monooleate.
