# Supplementary material for: Derivatives of Pyrimidine Nucleosides Affect Artificial Membranes Enriched with Mycobacterial Lipids
Source: Pharmaceutics. 2024 Aug 23;16(9):1110. doi: 10.3390/pharmaceutics16091110 (PMC11435317; doi:10.3390/pharmaceutics16091110)
Supplement: Supplementary file 1 [file pharmaceutics-16-01110-s001.zip › pharmaceutics-3144548-supplementary.pdf]

Supplementary Figure S1. The effects of different volume of DMSO on the ionic permeability of POPC (**a**), POPG (**b**), POPG/kMA (80/20 mol%) (**c**) and POPG/ThO (80/20 mol%) (**d**) bilayers bathed in 0.1 M KCl pH 7.4. The transmembrane voltage was 100 mV. Arrows indicate the moments of addition of the DMSO into the membrane bathing solution.

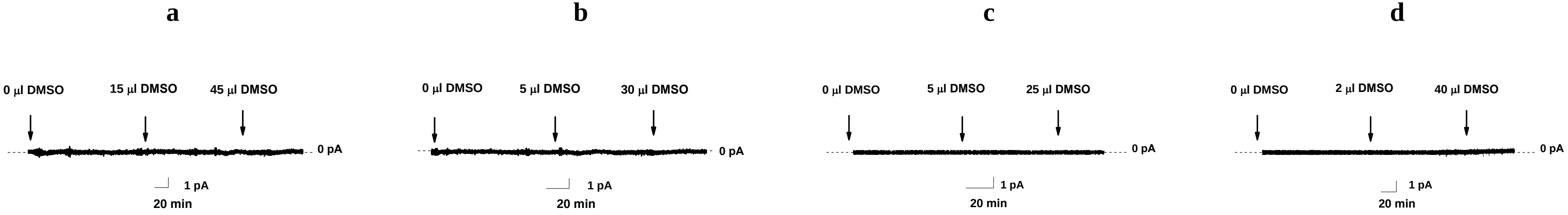

Supplementary Figure S2. Deconvolution analysis of the main peak on DPPC and DPPG thermograms in the presence various nucleoside derivatives at the lipid to agent ratio of 25:1, 10:1 and 5:1. The parameters characterizing distinct components are summarized in Supplementary Table S1.

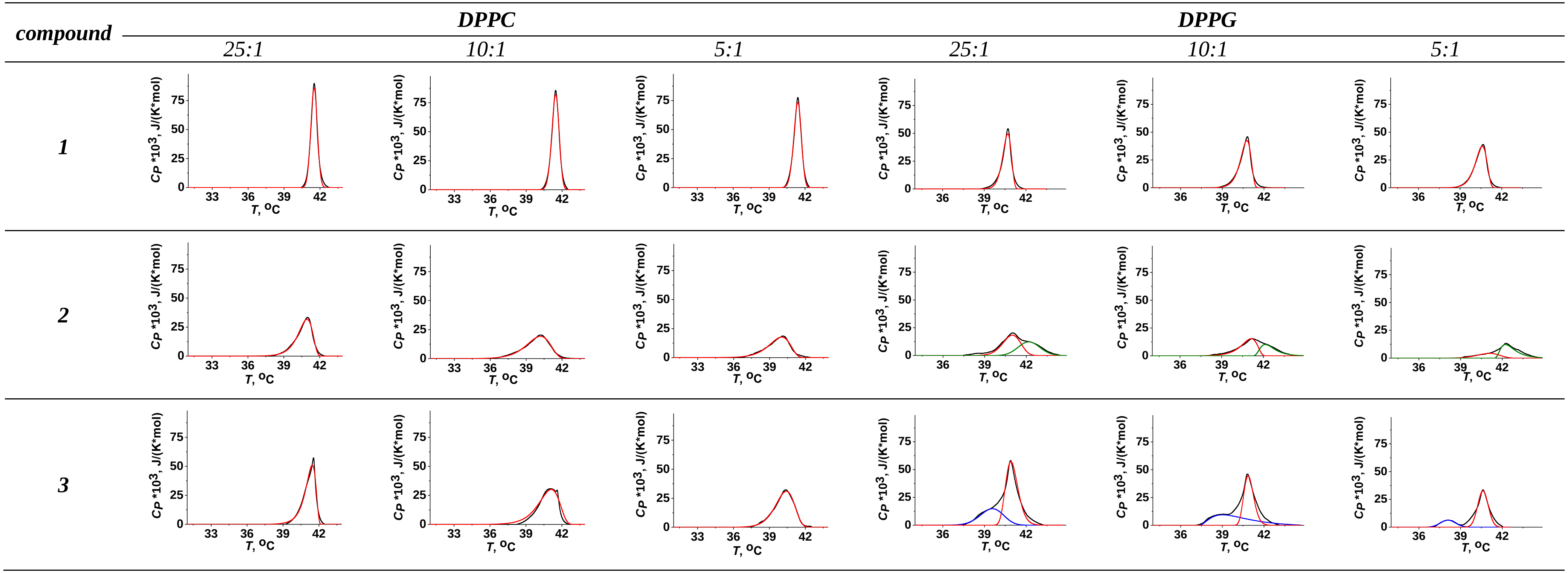

4

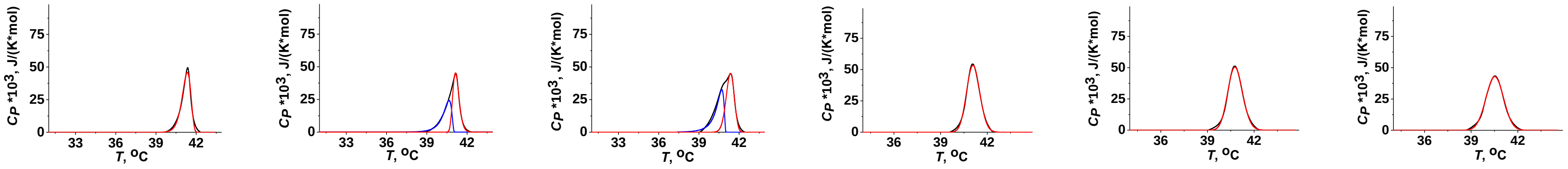
Cp *10^3, J/(K*mol)
T, °C

5

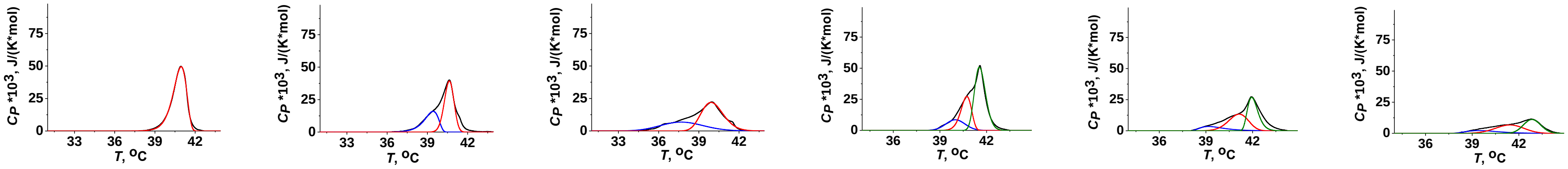
Cp *10^3, J/(K*mol)
T, °C

6

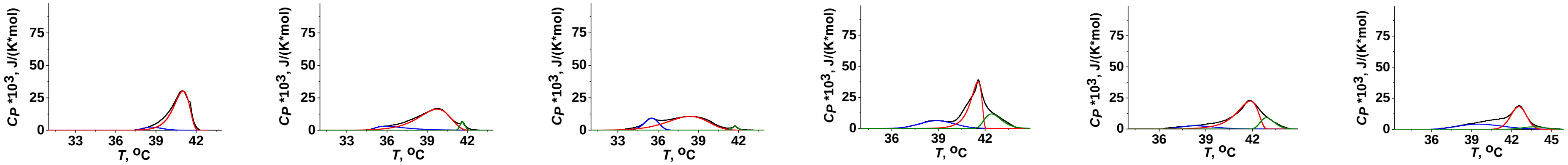
Cp *10^3, J/(K*mol)
T, °C

7

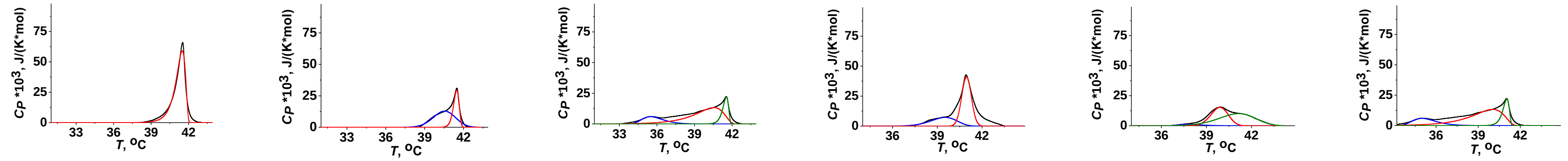
Cp *10^3, J/(K*mol)
T, °C

8

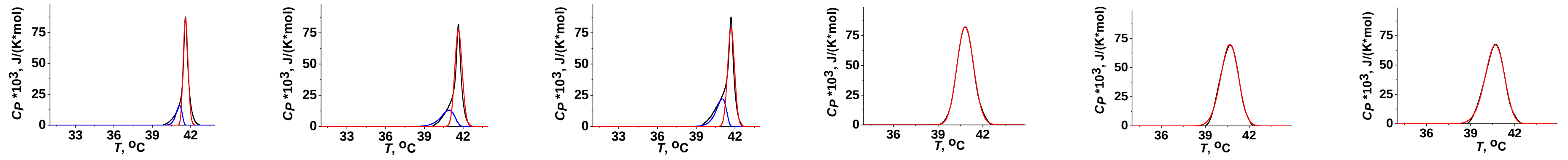
Cp *10^3, J/(K*mol)
T, °C

9

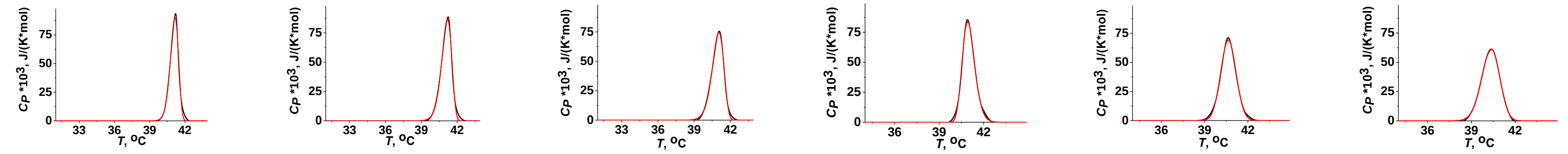
Cp *10^3, J/(K*mol)
T, °C

Supplementary Figure S3. The dependences of T_m-hysteresis of DPPC on the lipid:compound molar ratio. The dotted line represents the control level of T_m-hysteresis, which was observed in the absence of nucleoside derivatives.

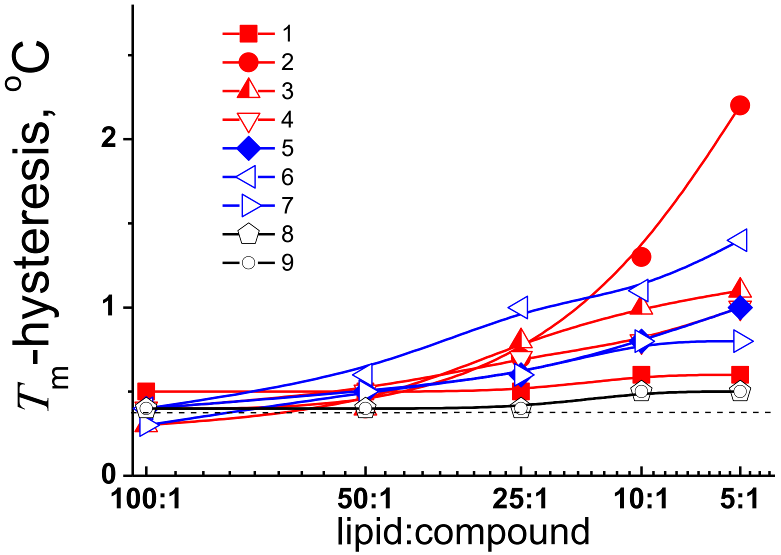

Supplementary Table S1. The main peak decomposition analysis in the presence of nucleoside derivatives

compound	***# of component***	T_{m_i}, °C								$\Delta H_i/\Delta H_{cal}$, %							
		DPPC				***DPPG***				***DPPC***				***DPPG***			
		0	*25:1*	*10:1*	*5:1*	*0*	*25:1*	*10:1*	*5:1*	*0*	*25:1*	*10:1*	*5:1*	*0*	*25:1*	*10:1*	*5:1*
1	#1	41.5	41.5	41.4	41.4	41.2	40.8	40.6	40.5	100	100	100	100	100	100	100	100
	#2		–	–	–		–	–	–		–	–	–		–	–	–
2	#1	41.6	40.9	40.3	40.1	41.4	40.9	41.0	41.1	100	100	100	100	100	55	52	34
	#2		–	–	–		42.2	42.3	42.3		–	–	–		45	48	66
3	#1	41.6	41.5	41.0	40.0	41.4	40.9	40.8	40.6	100	100	100	100	100	68	51	78
	#2		–	–	–		39.5	38.7	38.1		–	–	–		32	49	22
4	#1	41.5	41.2	41.1	41.1	41.4	41.0	40.8	40.5	100	100	56	54	100	100	100	100
	#2		–	40.6	40.5		–	–	–		–	44	46		–	–	–
5	#1	41.4	40.9	40.6	39.9	41.5	41.5	41.9	42.8	100	100	63	62	100	56	44	42
	#2		–	39.5	37.4		40.7	41.1	41.3		–	37	38		28	42	42
	#3		–	–	–		40.0	39.2	39.2		–	–	–		16	14	16
6	#1	41.6	41.1	41.6	41.7	41.3	42.5	42.9	43.8	100	91	5	3	100	24	18	9
	#2		38.7	39.7	38.3		41.5	41.8	42.4		9	80	75		52	70	56
	#3		–	35.9	35.5		38.8	38.3	39.1		–	15	22		24	12	35
7	#1	41.5	41.5	41.5	41.5	41.1	40.9	41.1	41.1	100	100	37	19	100	67	56	51
	#2		–	40.4	40.5		39.5	39.9	39.9		–	63	62		33	39	42
	#3		–	–	35.4		–	37.7	34.9		–	–	19		–	5	7
8	#1	41.5	41.5	41.5	41.5	41.4	40.8	40.7	40.6	100	76	74	70	100	100	100	100
	#2		41.1	41.0	40.9		–	–	–		24	26	30		–	–	–
9	#1	41.5	41.5	41.3	41.1	41.3	40.9	40.7	40.5	100	100	100	100	100	100	100	100

T_{m_i} is the temperature of maximum of *i* component of the main peak at the deconvolution;
$\Delta H_i/\Delta H_{cal}$ is the percentage contribution of the *i* component to the total area/enthalpy of the main peak.
